# Spontaneous Complete Remission of Acute Myeloid Leukemia in the Absence of Disease-Modifying Therapy following Severe Pulmonary Involvement by Coronavirus Infectious Disease-19

**DOI:** 10.1155/2022/2603607

**Published:** 2022-01-20

**Authors:** Maryam Barkhordar, Fatemeh Tajic Rostami, Marjan Yaghmaie, Mehrdad Abbaszadeh, Bahram Chahardouli, Seied Asadollah Mousavi

**Affiliations:** Hematology Oncology and Stem Cell Transplantation Research Center, Tehran University of Medical Sciences, Tehran, Iran

## Abstract

Coronavirus infectious disease-19 (COVID-19) usually alters the innate and adaptive immune setting by excessive production of proinflammatory cytokines, leading to a deviation in the natural course of simultaneous malignant disease. In the absence of disease-modifying therapy, complete remission of acute myeloid leukemia (AML) is an extraordinary event caused mainly by an immune-related mechanism secondary to a severe infectious process. We present a 57-year-old woman with a new diagnosis of AML associated with a 11q23/KMT2A abnormality who had achieved temporary spontaneous remission in the absence of disease-modifying therapy following the severe pulmonary infection with coronavirus lasting for six months. We review the literature and explain the potential impact of stimulated immune responses by COVID-19 on induction of remission in a patient with AML that could provide an excellent opportunity for new immune-based therapies to evolve for the hematologic malignancies. Despite the high ability of the immune process to destroy the malignant cells, the remission of duration is usually short. Therefore, it seems that continuing treatment after SR of AML by a consolidation regimen or bone marrow transplantation, based on a risk-adapted treatment approach, may reduce the recurrence risk.

## 1. Introduction

During the coronavirus infectious disease-19 (COVID-19) pandemic, concurrent diagnosis of COVID-19 with various hematologic and nonhematologic malignancies has been reported. Acute myeloid leukemia (AML) is a life-threatening hematologic cancer characterized by the monoclonal proliferation of myeloblasts to more than 20% of bone marrow cellularity.

The co-occurrence of AML and COVID-19 as a considerable therapeutic challenge has been described in multiple reports with fatal outcomes in some patients [1, 2]. The panel of international experts recommended holding the induction therapy of AML until the improvement of COVID-19-related symptoms and obtained a negative polymerase chain reaction (PCR) test [3].

Spontaneous remission (SR) in a newly diagnosed AML without disease-modifying therapy is a rare event described infrequently. According to the previous reports, SR of AML was usually described following recovery of a severe infection process; furthermore, we believe that immune activation secondary to an infectious process could eradicate the leukemic cells and induce a complete or partial remission [4, 7].

Herein, we present a new case of AML with 11q23/KMT2A abnormality who achieved complete molecular cytogenetic remission in the absence of disease-modifying therapy following recovery from COVID-19. To our knowledge, this is the first report of SR in a patient with AML and concomitant COVID-19. Besides, we explain the potential impact of immune-mediated mechanisms on induction of SR in a patient with AML infected with coronavirus, contributing to the further development of new immune-based therapies for the hematologic malignancies.

## 2. Case Report

A 57-year-old woman without any past medical history had presented with the petechial skin rash and bruising on the trunk and lower extremities and fatigue for about two weeks. The initial workup revealed anemia (Hb, 9.0 g/dL), thrombocytopenia (15 × 10^9^ platelets/L), and leukocytosis (13.4 × 10^9^ white blood cells (WBC)/L) in the complete blood count (CBC), and circulating blasts on the peripheral blood smear (PBS).

Therefore, she was referred to our center, a tertiary referral center in Tehran, with the impression of AML and admitted to the hematology ward on September 3, 2020. On physical examination, the positive findings were conjunctival pallor, scattered bruises, and petechial hemorrhage over the body surface and lower extremities. Lungs were clear on auscultation. According to the panel of international experts, we performed the screening tests to rule out COVID-19 [3] that demonstrated the normal findings for the spiral chest CT scan and negative report for the PCR test.

**Figure 1:**
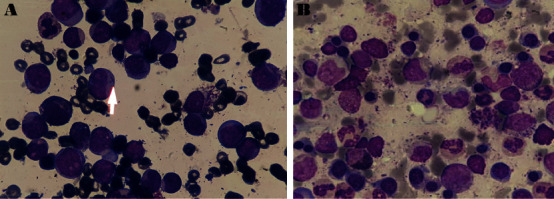
Bone marrow aspiration (BMA) morphology (1000 × Wright–Giemsa stain): (a) diagnostic BMA showing decreased trilineage hematopoiesis with a predominant medium-to-large-size myeloblast population with Auer rods in some blasts (arrow). (b) Follow-up BMA, after two months, showing maturing hematopoiesis with no abnormal myeloid blast population resembling complete remission.

After admission, BMA/B was performed and confirmed the initial diagnosis of AML M2 (Figure 1(a)). Pathologic evaluation revealed the 60% cellularity consisted of blast sheets occupying more than 65% of the bone marrow space with reduced megakaryocytes and scattered erythroid precursors. Immunophenotyping study by flow cytometry showed a predominant myeloblast population formed about 67% of the total cells with a positive expression of CD117, CD33, CD34, HLA-DR, and myeloperoxidase, aberrant expression of CD7, and negative for TdT, CD3, CD5, CD20, CD19, CD10, and CD14.

**Figure 2:**
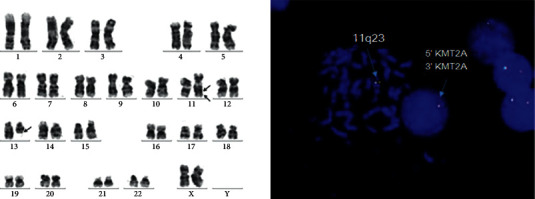
Karyotype: 46,XX,der (11) del (11) (q22 q23) ins (11; ?) (q22; ?), del(13) (q12q31) [50]. FISH (fluorescent in situ hybridization): nuc ish (KMT2A×1) (5′KMT2A con 3′KMT2A×1) [130/155], loss of 11q23.3 in 83% of cells.

Chromosomal evaluation of 50 metaphase cells by G-banded cytogenetic revealed an abnormal female karyotype with abnormality of chromosome 11q23 and insertion in the long arm of chromosome 11 (11q22) accompanied with deletion of the long arm of chromosome 13 in the same abnormal clone. Fluorescence in situ hybridization (FISH) analysis was performed and showed the “nuc ish (KMT2A×1) (5′KMT2A con 3′KMT2A×1) [130/155]” by using the KMT2A break-apart probe in 83% of cells, illustrated in Figure 2 Molecular analyses of aspiration samples were negative for NPM1, FLT-3 ITD, and FLT-3 TKD mutations. Overall findings confirmed the diagnosis of AML M2 with 11q23/KMT2A abnormality.

During the first few days of hospitalization and before starting the induction chemotherapy, she developed fever (38 °C), shortness of breath, cough, and hypoxemia. Coarse crackles were heard on auscultation of the lungs, and arterial blood gas analysis indicated pH 7.43, PaCO_2_ 42 mmHg, and PaO_2_ 46.7 mmHg on FiO_2_ of 40%. She received empirical therapy with meropenem and azithromycin followed by vancomycin and voriconazole for neutropenic fever.

**Figure 3:**
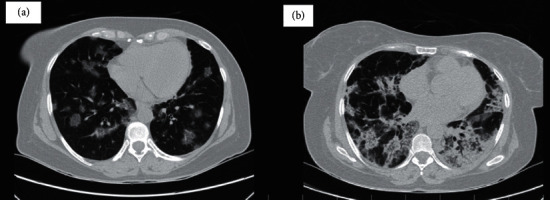
Spiral chest CT scans imaging: (a) diagnostic chest CT scan showing bilateral multifocal, dominantly peripheral, patchy ground-glass opacities suggestive of COVID-19 pneumonia. (b) Follow-up chest CT scan after 18 days showing notable progression in bilateral alveolar opacities with coarse reticular pattern (pulmonary fibrosis) in comparison with previous CT.

However, the pulmonary symptoms and hypoxemia became progressively worse, and blood culture samples were negative, which prompted us to rule out COVID-19 again. The second PCR for SARS-CoV-2 was performed, which turned positive, and a repeated CT scan showed bilateral and multifocal (dominantly peripheral) patchy ground-glass opacities suggestive COVID-19 (Figure 3(a)).

The patient was then transferred to the intensive care unit (ICU) on 13 September 2020 under the impression of COVID-19, received respiratory support by NIV (noninvasive ventilation), and was treated with remdesivir at 200 mg intravenously (IV) on the first day, followed by 100 mg daily for five days [8] and dexamethasone IV at 8 mg daily for ten days [9]. She was transfused by six units of packed red blood cells and ten units of platelets. Induction chemotherapy for AML was postponed to manage COVID-19 and achieve a negative PCR test [3].

After two weeks, she was returned to the hematology ward for starting the induction chemotherapy. However, she still required oxygen supplementation to maintain O_2_ sat >90%, and her general condition was not suitable for starting chemotherapy. The chest CT scan was repeated on 29.09.2020 and showed notable progression in bilateral alveolar opacities with a coarse reticular pattern compared with the previous CT scan (Figure 3(b)). Therefore, pirfenidone, colchicine (0.6 mg twice daily), and oxygen therapy with a reserve bag mask were prescribed by our pulmonologist under the impression of COVID-19-related pulmonary fibrosis.

While waiting to improve her respiratory function to make an appropriate therapeutic decision based on her new condition, the blood cell counts improved and dramatically returned to normal. Therefore, we repeated bone marrow sampling to reevaluate leukemia on 2 November 2020, two months after the initial diagnosis. BMA revealed a normocellular marrow with 55% cellularity contained less than 5% blast and trilineage maturation (Figure 1(b)), and flow cytometry showed about 3% myeloblast having the expression of CD34 and CD117.

The findings interestingly showed spontaneous morphologic remission in the absence of disease-modifying therapy of acute leukemia. Given these normal results, she was discharged without induction chemotherapy and underwent follow-up in an outpatient clinic. However, due to pulmonary complications caused by COVID-19, oxygen therapy at 1-2 L/min via nasal prongs was administered at home.

After four weeks of discharge on November 30, 2020, while the blood cell count was normal, another BMA/B was performed to evaluate the remission status that illustrated the normal cellularity and maturation with no evidence of abnormal blasts. To assess the measurable residual disease (MRD), we repeated the FISH analysis on the BMA sample by baseline probe of KMT2A, which revealed a normal result and proved the molecular cytogenetic remission. During follow-up, the patient's respiratory function improved so that the need for oxygen therapy was eliminated, and antifibrotic drugs (pirfenidone and colchicine) were discontinued.

Six months after the initial presentation, on Feb 2021, the surveillance marrow with normal blood counts revealed the normal trilineage hematopoiesis with no evidence of the leukemia relapse by morphologic assessment. Although, according to the genetic evaluation, loss of 11q23/KMT2A reappeared in 50% of cells by karyotyping and 21% of cells by FISH, in favor of molecular relapse; however, she refused a recommended treatment to prevent overt hematologic relapse.

During the next two months, thrombocytopenia and anemia recurred (WBC, 9 × 10^9^/L; Hb, 10 g/L; and PLT, 50 × 10^9^/L), and a subsequent marrow test, undertaken on April 2021, demonstrated more than 30% blast cells and confirmed the hematological recurrence of the primary disease.

**Figure 4:**
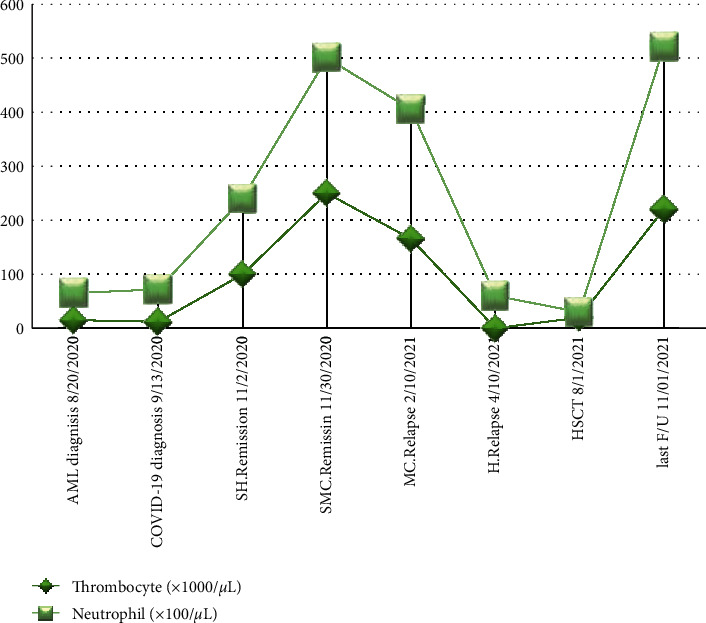
Blood cell changes and the time point of COVID-19 diagnosis, spontaneous remission, and relapse during the course of disease. SH.Remission, spontaneous hematologic remission; SMC.Remission, spontaneous molecular cytogenetic remission; H.Relapse, hematologic relapse; MC.Relapse, molecular cytogenetic relapse; HSCT, hematopoietic stem cell transplantation; and F/U, follow-up.

Therefore, the patient received the induction chemotherapy with cytarabine and daunorubicin (7 + 3) regimen and achieved a morphologic remission at BMA/B on day 28. In the second remission, she underwent allogeneic bone marrow transplantation from an HLA-matched sibling donor on 1 August 2021 using reduced-intensity conditioning with fludarabine and busulphan. Normal blood counts, morphologic remission, and full donor chimerism by short tandem repeat (STR) were achieved on day +28 after HSCT. At the last follow-up on day +90 after transplant, she was doing well, without any complication. The correlation between blood cells changes and the time point of COVID-19 diagnosis, spontaneous remission, and relapse are shown in Figure 4.

## 3. Discussion

We presented a new case of AML that the histologic diagnosis was confirmed by two subsequent examinations of the bone marrow. She achieved temporary SR in the absence of disease-modifying therapy after recovering from severe COVID-19, lasting six months. SR of AML is a relatively rare phenomenon in which the underlying mechanisms are not precisely known. Although based on existing published data, an immune-dependent mechanism secondary to the infectious process is considered the probable leading cause of SR [5–7], the other possible factors such as transfusion of blood products, corticosteroids, and colchicine that may contribute to the induction of remission in our case should be considered and discussed.

Colchicine is an alkaloid derivative of the plant (*Colchicum autumnale* L.), which has been known to possess a wide range of pharmacological activities, including antifibrotic, anti-inflammatory, and antiproliferative properties [10–12]. As an anti-inflammatory agent, colchicine is used to treat various inflammatory diseases such as gout, familial Mediterranean fever (FMF), and liver cirrhosis [11]. Colchicine has also been reported to exert an antiproliferative and anticancerous effect by mitotic arrest and cell death by inhibiting microtubules' formation, inducing oxidative stress, and increasing DNA damage [12].

Colchicine with pirfenidone was prescribed to our patient for the treatment of COVID-19-related pulmonary fibrosis [10] and was discontinued after two months. The first evidence of blood cell improvement appeared one month after starting colchicine. Therefore, the possible effect of colchicine in the induction of remission should be considered.

Blood product transfusion was discussed in some previous case reports as the possible cause of remission induction of acute leukemia in the absence of disease-modifying therapy and immunological mechanisms through the antileukemic effects of allogeneic lymphocytes, or inflammatory soluble cytokines are the probable underlying mechanism [13, 14]. Although our patient was transfused with low numbers of blood products before remission, the contribution of transfusion to the induction of remission could not be completely excluded.

In the current outbreak of COVID-19, corticosteroids have been widely used as anti-inflammatory agents that significantly reduce the complications among severe COVID-19 cases. Glucocorticoids are also an important part of the induction regimens for the lymphoid hematologic malignancies, but they have not a defined therapeutic role in myeloid leukemia. However, there is some evidence suggesting the possible efficacy of high-dose corticosteroids in inducing remission of AML in the absence of disease-modifying therapy [15, 16]. As our patient received low-dose dexamethasone for a short period, the impact of this agent to cause complete remission in our case seems unlikely but could not be excluded.

Rashidi et al. reported a case series study where 46 patients with SR of AML were reviewed. More than 70% of reported patients had confirmed pneumonia, soft-tissue infection, or bacteremia before SR [4]. According to the literature review, most patients did not receive any consolidation or maintenance therapy after achieving the SR, resulting in the experience of an early recurrence in most patients [4–7].

Theoretically, a standard immune system could potentially destroy the malignant cells by cellular and adaptive immune responses. Leukemia-derived dendritic cells (DCs) are the pivotal antigen-presenting cells (APCs) that initiate the antileukemic immune process by introducing the leukemia-associated antigens (LAAs) to the naïve T cells and stimulate the differentiation of naïve T cells into the CTLs [17]. DCs also stimulate the CTLs' and NK cells' activity to eradicate the leukemic cells by increasing the IFN-*γ*. A combination of cytokines including the granulocyte-macrophage colony-stimulating factor (GM-CSF), IL-4, TNF-*α*, IFN-*α*, IL-1*β*, IL-6, and PGE2 is necessary for converting the myeloid leukemia cells to the immature DCs and, subsequently, differentiation to the mature DCs [17–19].

However, the immune system is sometimes unable to destroy the abnormal cells, leading to various malignancies. Downregulation of MHC class II and costimulatory molecules on DCs can be responsible for antigen presentation impairment and antileukemic response failure [20]. The following possible mechanism is the immunosuppressive factors such as transforming growth factor-*β* (TGF-*β*), vascular endothelial growth factor (VEGF), and IL-10 that were secreted by leukemic cells [21].

On the other hand, some interfering events such as severe infection and excessive production of proinflammatory cytokines could alter the immune setting and overcome leukemia-induced immune suppression. The abovementioned process might lead to an antileukemia effect and even SR in the absence of disease-modifying therapy of acute leukemia described in some case reports [22].

COVID-19 usually affects both innate and adaptive immune responses. A large amount of chemokine is produced by DCs infected with the coronavirus [23]. Type 1 Interferon (IFN-*α*, *β*) and proinflammatory cytokines are secreted in the initial phases of the immune response. High plasma levels of G-CSF, IL-2, IL-7, TNF-*α*, and macrophage inflammatory protein 1 (MIP1) have been associated with severe lung damage in COVID-19 [24].

We introduced a case of AML with a 11q23/KMT2A abnormality that achieved SR in the absence of disease-modifying therapy lasting for six months following severe pulmonary involvement by COVID-19. Immune-dependent mechanisms secondary to COVID-19 can potentially destroy leukemic cells in addition to virus-infected cells by excessive production of proinflammatory cytokines. Indeed, SR of AML offers supporting evidence for the crucial role of a patient's immune responses to kill the leukemic cells and induction of remission; it also provides an excellent opportunity to evolve new immune-based therapies for the hematologic malignancies. However, other less-likely causes of remission induction, such as transfusion of blood products, corticosteroids, and colchicine, should also be considered.

Despite the high capacity of the immune process to eliminate the malignant cells, the spontaneous immune-related remission in the absence of disease-modifying therapy, similar to what happens following the induction chemotherapy, is usually transient. Therefore, it seems that continuing treatment after SR of AML by a consolidation regimen or bone marrow transplantation, based on the risk-adapted treatment approach and according to ELN 2016 risk categorization, may improve the outcomes and reduce their recurrence risk. Additional work is needed to determine the pathogenesis of immune-related remission in acute leukemia to optimize therapeutic strategies to treat the disease and improve the durability of disease control.

## Data Availability

The datasets generated during the current study are available from the corresponding author on a reasonable request.
